# Surface-exposed proteins of pathogenic mycobacteria and the role of cu-zn superoxide dismutase in macrophages and neutrophil survival

**DOI:** 10.1186/1477-5956-11-45

**Published:** 2013-11-28

**Authors:** Michael McNamara, Shin-Cheng Tzeng, Claudia Maier, Martin Wu, Luiz E Bermudez

**Affiliations:** 1 Department of Biomedical Sciences, Molecular and Cellular Biology Program, Corvallis, USA; 2 Department of Microbiology, Corvallis, USA; 3 Department of Chemistry, Corvallis, USA; 4 Oregon State University, Corvallis, Oregon 97331-4801, USA

**Keywords:** Mycobacterium avium, Surface-exposed proteome, Shotgun proteomics, Cu-Zn SOD

## Abstract

Pathogenic mycobacteria are important agents causing human disease. *Mycobacterium avium* subsp. *hominissuis* (*M. avium*) is a species of recalcitrant environmental pathogen. The bacterium forms robust biofilms that allow it to colonize and persist in austere environments, such as residential and commercial water systems. *M. avium* is also an opportunistic pathogen that is a significant source of mortality for immune-compromised individuals. Proteins exposed at the bacterial surface play a central role in mediating the relationship between the bacterium and its environment. The processes underlying both biofilm formation and pathogenesis are directly dependent on this essential subset of the bacterial proteome. Therefore, the characterization of the surface-exposed proteome is an important step towards an improved understanding of the mycobacterial biology and pathogenesis. Here we examined the complement of surface exposed proteins from *Mycobacterium avium 104*, a clinical isolate and reference strain of *Mycobacterium avium* subsp. *hominissuis.* To profile the surface-exposed proteins of viable *M. avium 104,* bacteria were covalently labeled with a membrane impermeable biotinylation reagent and labeled proteins were affinity purified via the biotin-streptavidin interaction. The results provide a helpful snapshot of the surface-exposed proteome of this frequently utilized reference strain of *M. avium.* A Cu-Zn SOD knockout mutant, MAV_2043, a surface identified protein, was evaluated regarding its role in the survival in both macrophages and neutrophils.

## Introduction

Pathogenic mycobacteria are responsible for a large number of human infections. The interaction between the pathogen and the human host is complex, but surface-exposed molecules are very significant in several aspects of the interaction.

An opportunistic pathogen, *Mycobacterium avium* subsp. *hominissuis* (*M. avium*) rose to prominence during the HIV/AIDS epidemic of the 1980’s and 1990’s [1]. *M. avium* is an environmental pathogen that is notable for its ability to form tenacious biofilms that allow it to persist and thrive in many environments [2]. The bacterium is also a capable pathogen that actively adheres to host tissue and invades host cells [3]. Following colonization, *M. avium* can survive and proliferate within the intra-cellular environment of host phagocytes, which it hijacks as vehicles for dissemination [4]. Significant progress has been made in the understanding of *M. avium* pathogenesis*,* but the mechanisms that the bacterium employs to sense and interact with its environment remain largely unknown. The characterization of the surface-exposed proteome *M. avium 104,* the primary reference strain for this species*,* is an important step in filling this gap.

The surface-exposed proteome of a bacterium is of great interest to both microbiologists and immunologists. Attachment, motility, molecular transport and conjugation are all functions that are critically dependent on proteins exposed at the surface interface. In addition, surface-expressed proteins are likely to be important to interaction with phagocyte cells [5,6]. Surface proteins are also primary targets for both innate and adaptive immune responses. Effective engagement of pathogens by the immune system requires the recognition of accessible targets, which tend to be surface-exposed molecules. Accordingly, surface-exposed proteins are disproportionately represented in the antigenic profiles from mycobacteria-infected animal hosts [7,8].

There are several challenges that complicate the analysis of surface-exposed proteins. The biggest obstacle is often the selective isolation of these proteins, which represent a small fraction of the total cellular proteome. The covalent labeling of surface-exposed proteins with affinity tags using membrane-impermeable reagents, particularly biotin-based reagents, is a proven method for this type of analysis [9-11]. The primary advantage of biotinylation is the ability to selectively label surface-exposed proteins in mild, isotonic buffers. A unique challenge of this standard approach is the effective solubilization of target proteins, in buffers compatible with affinity purification, from the mycomembrane of *M. avium,* which is a durable, cross-linked and hydrophobic structure [12]. To address this issue this study utilized two experimental protein extraction buffers that are optimized be used at two different concentrations for protein extraction and affinity purification, respectively. At full strength, both buffers are effective at solubilizing and/or denaturing the total protein from whole cell lysates. Subsequently, non-solubilized debris is then removed and the samples are diluted in order to be compatible with biotin-streptavidin based affinity purification. Following purification, we employed on-bead digestion in tandem with “shotgun” mass spectrometry to characterize the surface-exposed proteome of our target bacteria, *M. avium* subsp. *hominissuis*. In total, this analysis yielded a detailed profile of the surface proteome of a clinically-relevant strain of mycobacteria, *M. avium 104*. It also offers potential targets for attenuation of the bacterial virulence as demonstrated in this study.

## Materials and methods

### Preparation of M. avium cultures

*M. avium 104* (a clinical isolate and sequenced reference strain of *Mycobacterium avium* subsp. *hominissuis*) was grown on Middlebrook 7H10 agar and transferred into 200 ml of Middlebrook 7H9 broth supplemented with 10% oleic albumin dextrose catalase (OADC) (Hardy Diagnostics, Santa Maria CA) and cultured at 37°C with constant agitation. Exponentially growing bacteria were harvested by centrifugation (1,500 × *g* for 15 min) and washed twice with WB-PBS (150 mM NaCl, 20 mM Na_2_HPO_4_, .05% Tween-20 (vol/vol), pH 7.2) and twice with BupH-PBS (150 mM NaCl, 100 mM Na_2_HPO_4_, pH 7.3). Bacteria were then separated into equal aliquots (approximately 100 mg of bacteria per aliquot), pelleted and re-suspended in 1 ml BupH-PBS.

*M. avium* transposon library was created as reported previously [13] and the mutant in the MAV_2043 gene was sequenced as reported [13]. The mutation had no effect on the ability of the clone to grow in vitro.

### Biotinylation M. avium surface-exposed proteins

Immediately prior to biotinylation, a fresh solution of Sulfo-NHS-LC-Biotin (LC-Biotin) (Pierce, Rockford IL) was prepared at a concentration of 1 mg/ml in BupH-PBS. For experimental samples, 500 μl of either reagent solution was added per aliquot, for a total reaction volume of 1.5 ml. For negative controls (no biotin), 500 μl of BupH-PBS was added instead. The biotinylation reaction was allowed to proceed for 20 min at 23°C with gentle agitation. Upon completion of the labeling reaction, each aliquot was washed twice with BupH-PBS supplemented with glycine (10 mg/ml) and twice with plain BupH-PBS to inactivate and remove any unbound biotinylation reagent. Bacterial samples were then pelleted and the supernatant was discarded.

### Total protein extraction

Bacterial samples were placed on ice and 300 μL of 100 μm glass beads (Sigma, St. Louis, MO) were added to each sample. Two protein extraction buffers were used in this study: Detergent Extraction Buffer (DEB) (150 mM NaCl, 20 mM Na_2_HPO_4_, 0.05% Tween-20 (vol/vol), 0.1% Triton X-100 (vol/vol), 0.2% CHAPS [wt/vol], pH 7.3) and Urea Extraction Buffer (UEB) (150 mM NaCl, 20 mM Na_2_HPO_4_, 0.05% Tween-20 (vol/vol), 7 M urea, 0.2% CHAPS [wt/vol], pH 7.3). Pellets of biotin-labeled *M. avium* were resuspended in 700 μl of either DEB or UEB. Bacteria were disrupted by bead milling (6 pulses of 30 s). After disruption, samples were centrifuged (12,000 × *g* for 15 min) to pellet non-soluble components and supernatant was removed to a clean tube. Each sample was subjected to two rounds of protein extraction, and the resulting supernatants were pooled for a final sample volume of ~1.2 ml.

### Analysis of endogenous biotinylation and solubilization capacity of protein extraction buffers

To assess endogenous biotinylation and to compare the relative protein solubilization capacity of the buffers used in this study, total protein was extracted using each buffer from both biotinylated and non-biotinylated aliquots of *M. avium*. Briefly, total protein from 4 aliquots of non-biotinylated *M. avium* and four aliquots of Sulfo-NHS-LC-Biotin-labeled bacteria were extracted in either BupH-PBS, WB-PBS, DEB or UEB. Bacteria were disrupted with bead milling and soluble protein was isolated, as described above. Equal parts of each protein sample were separated by SDS-PAGE. Following SDS-PAGE separation, proteins were transferred to wet nitrocellulose membranes and analyzed by Western blot. IRDye-680 streptavidin (Licor, Lincoln, NE) was used to probe membranes for biotinylated proteins, following manufacturer protocol. Biotinylation patterns were visualized on an Odyssey Scanner (Licor).

### Affinity purification with streptavidin-coupled Dynabeads

Prior to affinity purification, 120 μl aliquots of magnetic, streptavidin-coupled C1 Dynabeads (Invitrogen, Carlsbad, CA) were washed twice with WB-PBS using a magnetic stand. Also prior to affinity purification, each sample was diluted with 3 volumes of WB-PBS. Diluted buffers were used for subsequent affinity purification and washing steps ((DEB (dilute): 150 mM NaCl, 20 mM Na_2_HPO_4_, 0.05% Tween-20 (vol/vol), 0.025% Triton X-100 (vol/vol), 0.05% CHAPS [wt/vol], pH 7.3) and (UEB (dilute): 150 mM NaCl, 20 mM Na_2_HPO_4_, 0.05% Tween-20 (vol/vol), 1.75 M urea, 0.05% CHAPS [wt/vol], pH 7.3)). Each diluted protein sample was mixed with a 120 μl aliquot of Dynabeads and incubated for 60 min at 23°C with gentle agitation. After incubation, samples were washed three times with their respective affinity capture buffer (either DEB (dilute) or UEB (dilute)). Samples were then washed twice with WB-PBS and three times with ammonium bicarbonate buffer (ABB) (50 mM NH_4_HCO_3_, pH 7.8). Each sample was then resuspended in ABB and split into two equal aliquots. Two of these aliquots were used for enzymatic proteolysis and the third was used to visualize the captured proteins by SDS-PAGE.

### Enzymatic digest of purified surface proteins

Prior to enzymatic digestion, aliquots of Dynabeads, complexed with captured proteins, were resuspended in 50 μl of ABB supplemented with 0.025% [wt/vol] ProteaseMax (Promega, Madison WI) a surfactant showed to increase the efficiency of trypsin digestion, and incubated at 37°C with constant agitation for 20 min. Then, 46 μl of ABB and 1 μl of 500 mM dithiothreitol (Sigma) was added to each aliquot, and samples were incubated at 60°C for 20 min. Next, 3 μl of 500 mM iodoacetamide (Sigma) was added to each aliquot and samples were incubated in darkness for 15 min at 23°C. Five μl of acetonitrile (ACN) was added to each aliquot and samples were incubated at 37°C for 5 min. For enzymatic proteolysis, each equal aliquot was digested with 1 μg of either Trypsin Gold or Glu-C (Promega) for 6 h at 37°C, with constant agitation. Following proteolysis, the Dynabeads were removed from the LC-Biotin-treated samples with a magnetic stand. Peptides from each aliquot were purified and desalted on C-18 reverse phase spin columns (Sartorius, Goettingen Germany), according to manufacturer instructions. Following purification, samples were dried by vacuum centrifugation and resuspended in 8 μl of MS Loading Buffer (95% H_2_0, 4.9% ACN (vol/vol), 0.1% formic acid (vol/vol)).

### Preparation of negative controls

To detect non-specific background and endogenously biotinylated proteins, samples of *M. avium* were isolated from the conditions described above for use as negative controls. These samples were processed in the previously described manner, except with biotinylation omitted. Data from negative controls were pooled to create a master list of false positive identification and these proteins were then subtracted from the experimental data sets.

### LC-MS/MS analysis

Data dependent LC-MS/MS analyses were performed on LTQ-FT Ultra mass spectrometer with IonMax ion source (Thermo) coupled to a nanoAcquity Ultra performance LC system (Waters) equipped with a Michrom Peptide CapTrap column and a C18 column (Agilent Zorbax 300SB-C18, 250 × 0.3 mm, 5 μm). A binary gradient system was used consisting of solvent A (0.1% aqueous formic acid) and solvent B (ACN containing 0.1% formic acid). Two μl of C-18 column purified peptides were then trapped and washed with 3% solvent B at a flow rate of 5 μl/min for 3 min. Trapped peptides were then eluted on to analytical column using a linear gradient from 3% B to 30% B at a flow rate of 4 μl/min over 35 min. Column was maintained at 37°C during the run. The mass spectrometer was operated in a data-dependent acquisition mode. A full FT-MS scan (m/z 350-2000) was alternated with CID MS/MS scans of the five most abundant doubly- or triply-charged precursor ions. As the survey scan was acquired in the ICR cell, the CID experiments were performed in the linear ion trap where precursor ions were isolated and subjected to CID in parallel with the completion of the full FT-MS scan. CID was performed with helium gas at a normalized collision energy 35% and activation time of 30 ms. Automated gain control (AGC) was used to accumulate sufficient precursor ions (target value, 5 × 10^4^/micro scan; maximum fill time 0.2 s). Dynamic exclusion was used with a repeat count of 1 and exclusion duration of 60 s. Data acquisition was controlled by Xcalibur (version 2.0.5) software (Thermo).

### Database search

Thermo RAW data files were processed with Proteome Discoverer version 1.2 using default parameters. A Mascot (version 2.2.04) search against whole SwissProt 2010 database (523151 sequences; 184678199 residues) or a *Mycobacterium avium* (strain 104) database (obtained from UniProt; 5040 sequences; 1586464 residues) was launched from Proteome Discoverer with the following parameters: the digestion enzyme was set to Trypsin/P and two missed cleavage sites were allowed. The precursor ion mass tolerance was set to 5 ppm; whereas, fragment ion tolerance of 0.8 Da was used. Dynamic modifications included carbamidomethyl (+57.0214 Da) for Cys and oxidation (+15.9994 Da) for Met. Lists of identified proteins from each sample were summarized by Scaffold 3 (Proteome Software, Portland OR). Inclusion of proteins in the final data set required at least two unique peptide identifications per protein and a minimum protein identification probability of 95%, as calculated by Scaffold 3.

### Phagocytes

Human monocyte-derived macrophages were purified from buffy-coat as previously described [14]. Monocytes were seeded in a 24 well tissue culture plate in presence of RPMI-1640 supplemented with 5% fetal bovine serum and allowed to mature to macrophage in 3 days. Monolayers (10^5^ cells) were infected with *M. avium* MAV_2043 Cu-Zn SOD KO and with *M. avium* MAC104 wild type (5 × 10^5^ cells). Monolayers were washed after 30 min and the number of intracellular bacteria determined after lysis of the monolayer [14] at 1 and 2 hours after infection and plating onto 7H10 agar plates.

Neutrophils were purified from buffy-coat as previously reported [15]. They were maintained on suspension in RPMI-1640 plus 5% FBS. Neutrophils (10^5^ cells) were then infected with *M. avium* MAV_2043 KO or MAC104 wild type (10^5^ cells) for 30 min under constant rotation. After the neutrophils were centrifuged at 500 rpm for 10 min and lysed to quantify viable intracellular bacteria. The eukaryotic cell lysate was plated onto 7H10 agar plates.

*E. coli* HB101, cultured in LB medium was used as control.

### Statistics

Comparison between experimental groups was carried out using the Student’s T test. A value of p < 0.05 was considered to be significant.

## Results and discussion

### Comparison of protein extraction and affinity purification buffers

**Figure 1:**
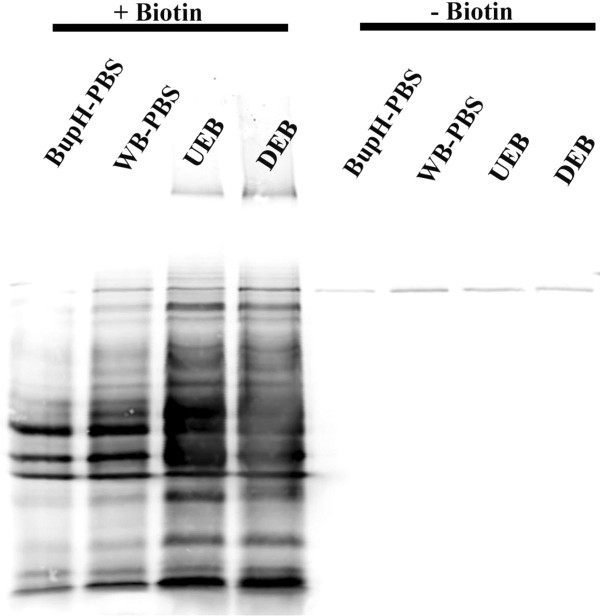
**Western blot analysis (anti-biotin) of total protein extracted from both Sulfo-NHS-LC-Biotin labeled *M. avium *(right) and unlabeled *M. avium *(right)*.*** All four buffers used in this study (BupH-PBS, WB-PBS, UEB and UEB) were compared to analyze their capacity to solubilize labeled proteins. Unlabeled *M. avium* samples (right) illustrate the relatively low levels of endogenous biotinylation.

**Figure 2:**
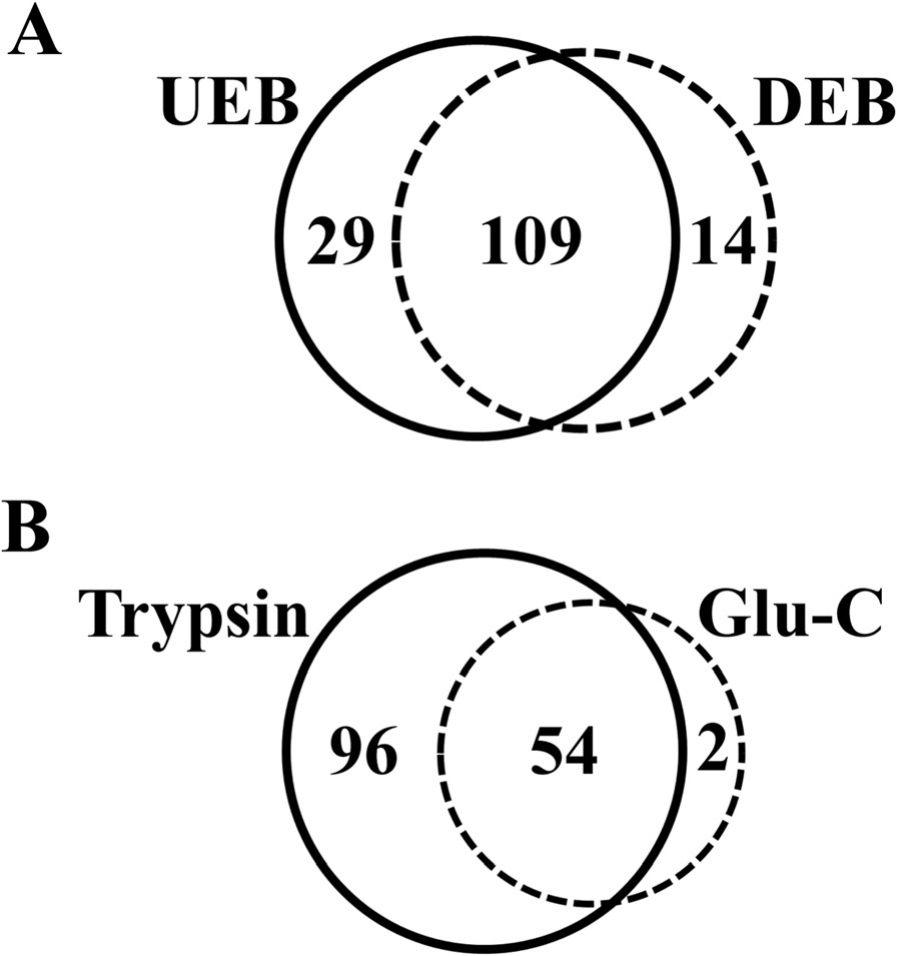
**Venn diagram of proteins detected in each experimental condition.** Values indicate total number of surface-exposed proteins detected in each experimental condition, with overlap indicating those proteins detected in both conditions. **A.** Comparison of extraction and purification buffers; surface proteins detected from samples extracted and purified with either Urea Extraction Buffer (UEB) or Detergent Extraction Buffer (DEB). **B.** Comparison of proteolytic enzymes; surface proteins detected from samples digested with either Trypsin or Glu-C proteases.

Prior to initiating this study, several potential buffer components were tested for their capacity to solubilize mycobacterial proteins and their compatibility with the streptavidin-biotin affinity interaction. The results of these experiments indicated that both non-ionic detergents (Triton X-100 and Tween-20) and urea (7 M) were reasonably effective for total protein solubilization. However, this analysis also indicated that the concentrations of detergents and/or chaotropic reagents that yielded maximum protein solubilization were deleterious to the streptavidin-biotin affinity interaction. To resolve this conflict, we employed a strategy of protein extraction at high detergent/urea concentrations followed by dilution prior to affinity purification. In this study we evaluated two buffer mixtures that had shown promise in earlier testing, a detergent-based extraction buffer (DEB) and a urea-based extraction Buffer (UEB). Analysis of the protein extraction capacity of both of these buffers indicated that they were similarly effective at solubilizing total protein (Figure 1). With respect to the complement of proteins that were detected in each buffer condition, our analysis indicated that the large majority of the observed proteins were the same between both buffers (Figure 2A).

### Comparison of trypsin and Glu-C proteolysis

Trypsin, which cleaves after lysine and arginine residues, is the protease most commonly used to digest proteins into peptides for identification with mass spectrometry. However, several proteins that are known to be surface-exposed in mycobacteria (e.g. many of the PPE and PE family proteins) are relatively lysine and arginine poor [16]. Because of this limitation, we hypothesized that the addition of an alternative proteolytic enzyme may be beneficial, increasing the number of identified proteins and the peptide coverage of those proteins. In this study, we compared the data generated by trypsin proteolysis with that generated by Glu-C proteolysis, which cleaves after glutamic acid residues (at pH 8). As expected, proteolysis with Glu-C generated fewer overall peptides, and the majority of the corresponding proteins were also identified in the trypsin samples (Figure 2B). However, the peptides generated by Glu-C digestion were highly complementary with those produced by trypsin digestion and significantly improved the peptide coverage of identified proteins. Additionally, several proteins that were lysine and arginine poor were uniquely identified by Glu-C analysis (Additional file 1: Table S1).

### Observed surface proteins

Across all of the experimental conditions, a total of 152 putative surface-exposed proteins from *M. avium 104* were detected (Additional file 1: Table S1). The vast majority of these proteins were detected in both experimental buffer systems, suggesting a similar complement of proteins were solubilized (Figure 2). As expected, many proteins with putative roles in the biogenesis of the mycomembrane were observed. A particularly interesting group of proteins are those that are thought to be directly associated with mycobacterial virulence (Table 1). Many of these proteins are conserved homologs of mycobacterial antigens that are known to be surface-exposed in other species of mycobacteria [8,17-19]. In fact, one of them, Cu-Zn SOD is known to be a virulence associated protein in many pathogens but its role in mycobacteria has not been fully studied.

**Table 1:** **Virulence-associated proteins observed on the surface of *M. avium 104***

**Uniprot annotation**	**Accession**	**Gene**	**Reference**
Superoxide dismutase [Mn]	A0Q988_MYCA1	MAV_0182	[20]
Antigen 85A	A0Q9C0_MYCA1	MAV_0214	[21]
Antigen 85C	A0Q9C1_MYCA1	MAV_0215	[21]
ABC transporter dppD	A0QA11_MYCA1	MAV_0467	[22]
Lipoprotein LpqE	A0QAB1_MYCA1	MAV_0569	[23]
Mce family protein	A0QBC2_MYCA1	MAV_0949	[24]
Superoxide dismutase [Cu-Zn]	A0QEC3_MYCA1	MAV_2043	[25]
Wag31 protein	A0QF61_MYCA1	MAV_2345	[26]
Antigen 85B	A0QGG5_MYCA1	MAV_2816	[21]
ModD protein	A0QGK7_MYCA1	MAV_2859	[27]
NlpC/P60 family protein	A0QHK2_MYCA1	MAV_3208	[28]
LprG protein	A0QI11_MYCA1	MAV_3367	[29]
Protein export protein SecF	A0QIB1_MYCA1	MAV_3467	[30]
Protein export protein SecD	A0QIB2_MYCA1	MAV_3468	[30]
Heparin binding hemagglutinin	A0QLL5_MYCA1	MAV_4675	[31]
MVIN family protein	A0QNC0_MYCA1	MAV_5298	[32]

Another group of interesting proteins are those associated with the mycobacterial Type 7 Secretion System (T7SS), which is known to export a range of proteins necessary to mycobacterial virulence [33]. *Mycobacterium avium* has four loci that encode for distinct copies of this unique secretion system [16]. Furthermore, the secretion apparatus encoded by each locus is believed to be responsible for the export of a relatively unique set of substrate proteins. This analysis detected evidence of structural proteins associated with three of the four ESX loci (Table 2). In addition, several putative substrate proteins from the PPE and PE protein families were also observed, although nearly all of these were associated with one ESX loci, ESX-5.

**Table 2:** **Type seven secretion system (T7SS) associated proteins observed on the surface of *M. avium 104***

**Uniprot annotation**	**Accession**	**Gene**	**T7SS Loci**
Uncharacterized protein	A0Q962_MYCA1	MAV_0156	ESX-2
Conserved membrane protein	A0Q965_MYCA1	MAV_0159	ESX-2
PPE family protein	A0QGQ3_MYCA1	MAV_2905	ESX-5
PPE family protein	A0QGQ4_MYCA1	MAV_2906	ESX-5
PPE family protein	A0QGQ7_MYCA1	MAV_2909	ESX-5
PPE family protein	A0QGQ8_MYCA1	MAV_2910	ESX-5
Uncharacterized protein	A0QGR5_MYCA1	MAV_2917	ESX-5
Secretion protein	A0QGR7_MYCA1	MAV_2919	ESX-5
PE family protein	A0QGS1_MYCA1	MAV_2923	ESX-5
Uncharacterized protein	A0QGT1_MYCA1	MAV_2933	ESX-5
PPE family protein	A0QKH6_MYCA1	MAV_4274	Unknown
Uncharacterized protein	A0QKS7_MYCA1	MAV_4380	ESX-4
Serine esterase	A0QKU1_MYCA1	MAV_4394	ESX-4

### Evaluation of the mutation in a surface protein, MAV_2043

Monocyte derived macrophages were infected with the wild type and MAV_2043, and number of intracellular viable bacteria were determined after 1 and 2 h. As shown in Table 3, the deficiency in MAV_2043 (Cu-Zn SOD) had a small effect on the survival of *M. avium* in macrophages. However, when the host cells were neutrophils, the absence of superoxide dismutase on the surface of *M. avium* was associated with significant decrease in bacterial viability (Table 4).

**Table 3:** **Role of Cu-Zn SOD of *M*. *avium *** **in human monocyte-derived macrophage infection**

**CFU/10**^**5 **^**macrophage lysate**			
Infection	30 min	1 h	2 h
WT *M. avium* 104	3.4 ± 0.3 × 10^4^	2.8 ± 0.4 × 10^4^	2.9 ± 0.2 × 10^4^
MAV_2043 KO *M. avium* 104	3.4 ± 0.4 × 10^4^	2.9 ± 0.2 × 10^4^	2.9 ± 0.4 × 10^4^
*E. coli* HB101	6.1 ± 0.3 × 10^4^	8.5 ± 0.3 × 10^3(1)^	6.1 ± 0.3 × 10^3(1)^

(1) p < 0.05 compared to 30 min time point.

**Table 4:** **Role of Cu-Zn SOD of *M*. *avium *** **in neutrophil infection**

**CFU/10**^**5 **^**neutrophil lysate**		
Infection	30 min	1 h
WT *M. avium* 104	4.3 ± 0.4 × 10^3^	4.6 ± 0.3 × 10^3^
MAV_2043 KO *M. avium* 104	2.6 ± 0.5 × 10^3^	5.1 ± 0.4 × 10^2(1)^
*E. coli* HB101	7.4 ± 0.3 × 10^4^	6.8 ± 0.3 × 10^2(1)^

(1) p < 0.05 compared with the number of bacteria at 30 min.

Our results with *M. avium* resembles the results obtained with *Mycobacterium tuberculosis* and macrophages [34] in which the deficiency of Cu-Zn SOD led to a small decrease in the ability to survive although the authors did not evaluate the interaction between *M. tuberculosis* and neutrophils. The study also adds to previous observation that neutrophils can kill *M. avium* in vivo [15,34,35] and seem to be part of the effective innate response against *M. avium*[15,36]. Neutrophils are capable of producing and releasing increasing amounts of superoxide anion than macrophages. During virulent *M. avium* infection, neutrophils appear to be important, but their role is only in the initial phase of the infection, which suggests that *M. avium* avoids them and preferentially infects macrophages (observation not shown). Recently, we demonstrated that *M. avium*, when phagocytosed by macrophages, expresses additional proteins in the surface [37], but MAV_2043 is observed on the surface even before contact with phagocytic cells.

### Potential contaminants

A number of proteins were detected that are possible contaminants, including ribosomal proteins, DNA gyrase and other nucleotide binding proteins (Additional file 1: Table S1). Although nucleotide proteins have been previously observed in the mycomembrane and at the surface of several species of *Mycobacterium*, these proteins may indeed represent persistent contaminants [18,38,39]. Because nucleotide binding proteins tend to have abundant arginine and lysine residues, they are excellent substrates for trypsin-based digestion. Additionally, contaminating DNA may cause many of these proteins to be non-specifically co-precipitated along with labeled proteins. Ultimately, the proteins detected in this study should be considered a preliminary profile, pending the independent confirmation of these observations with complementary methods.

## Conclusions

The selective biotinylation of surface-exposed proteins using membrane impermeable reagents has become an important tool in the study of this subset of the cellular proteome. This study adapted the aforementioned method to investigate the surface proteins *M. avium 104*. We demonstrated the feasibility of this specific approach by characterizing the surface proteome of a reference strain of *M. avium 104.* We also confirmed the important role of one of the surface associated proteins, Cu-Zn superoxide dismutase While its participation on macrophage infection was limited, its value to *M. avium* upon infection of neutrophils, what happens during the initial phase of the infection in vivo [15], was significant. The method used for determination of surface proteome then can identify important virulence factors in bacterial pathogens.

## Ethical approval

The research reported was approved by the University Biosafety Committee.

## Competing interests

The authors declare that there are no competing interests.

## Authors’ contributions

MMcN: Design, perform experiments, analyzed results, wrote the paper. S-CT: Perfom proteome assays, analyzed results. CM: Perform proteome assays, analyzed results. MW: Evaluated the mutants. LEB: Design experiments, analyzed results, edited the paper. All authors read and approved the final manuscript.

## Supplementary Material

Additional file 1: Figure S1 List of M. avium surface proteins obtained by Mass-Espec. Click here for file
